# Substance use in sexual minority youth: prevalence in an urban cohort

**DOI:** 10.1186/s13034-023-00657-0

**Published:** 2023-09-16

**Authors:** Florian Vock, Lydia Johnson-Ferguson, Laura Bechtiger, Niklaus Stulz, Joh von Felten, Manuel Eisner, Urs Hepp, Denis Ribeaud, Lilly Shanahan, Boris B. Quednow

**Affiliations:** 1 Swiss AIDS Federation, Zurich, Switzerland; 2 Jacobs Center for Productive Youth Development, University of Zurich, Zurich, Switzerland; 3 Department of Psychology, Clinical and Developmental Psychology, University of Zurich, Zurich, Switzerland; 4 Experimental and Clinical Pharmacopsychology, Department of Psychiatry, Psychotherapy and Psychosomatics, Psychiatric Hospital of the University of Zurich, Zurich, Switzerland; 5 Integrated Psychiatric Services Psychiatry Winterthur – Zürcher Unterland, Winterthur, Switzerland; 6 Milchjugend, LGBTQ+ Youth Organization, Zurich, Switzerland; 7 Institute of Criminology, University of Cambridge, Cambridge, UK; 8 Department of Consultation-Liaison Psychiatry and Psychosomatic Medicine, University Hospital Zurich, University of Zurich, Zurich, Switzerland

**Keywords:** Sexual minority youth, Adolescents, Drug use, Addiction, LGBTQ

## Abstract

**Background:**

Little comparative data on substance use (SU) between sexual minority youth (SMY) and heterosexual youth (HET) is available. This study compares the prevalence of SU in an urban cohort between SMY and HET and evaluates demographic and psychosocial predictors of SU.

**Methods:**

Data came from a prospective-longitudinal cohort study in an urban setting (N = 1297). SU and psychosocial variables such as internalizing symptoms, self-control, sensation-seeking, bullying-victimization, subjective stress, leisure activities, and peer influences were assessed with self-reports at age 17 and 20. SU was stratified by sex and sexual attraction, and the groups were compared using regression models, with demographic and psychosocial variables included as covariates.

**Results:**

SMY- and HET-youth displayed differences in a number of psychosocial variables. Overall, SMY- and HET-youth differed in their 12-months prevalence of SU: At age 17, SMY-females had significantly higher rates of SU than HET-females for cannabis (aOR = 2.14, p = 0.04), ecstasy/MDMA (aOR = 4.29, p = 0.01), and hallucinogens (aOR = 5.59, p = 0.02). At age 20, SMY-females had significantly higher rates of SU than HET-females for tobacco (aOR = 2.06, p = 0.03), cannabis (aOR = 2.24, p = 0.004), ecstasy/MDMA (aOR = 3.93, p < 0.001), stimulants (aOR = 3.45, p = 0.002), and hallucinogens (aOR = 6.65, p < 0.001). SMY-males reported significantly lower rates for tobacco and cannabis than HET-males at age 17. At age 20, they reported significantly higher rates for the use of ecstasy/MDMA (aOR = 2.30, p = 0.04) and hallucinogens (aOR = 2.43, p = 0.03).

**Conclusions:**

Given that psychosocial variables were significant covariates of SMY-status and SU, our results underline the importance of accounting for these when explaining differences in SU between adolescents. While differentiation by sex is established in most studies, such standardized comparisons are lacking with regards to sexual identities. But knowledge about SU of SMY is critical for designing effective interventions. This is especially true for SMY-females: Thus, SU in SMY-females early in life needs to be explored more thoroughly and addressed with adequate prevention measures.

**Supplementary Information:**

The online version contains supplementary material available at 10.1186/s13034-023-00657-0.

## Background

Previous studies have highlighted higher rates of substance use (SU) of lesbian, gay, bisexual, transgender, and queer (LGBTQ) adolescents compared to heterosexual youth in various countries [24, 26, 32, 34]. Data from the 2015 Global Drug Survey showed significantly higher rates of lifetime, 12-month, and past-month SU of sexual minority youth than heterosexuals on all 11 illicit substances that were examined [11].

SU bears the risk of the development of substance use disorders (SUD) and a range of negative health outcomes [9, 16]. For example, a study with the same cohort analyzed in this publication found that frequent teenage cannabis use was associated with more problematic SU, more delinquency, and lower functional well-being at age 20 [47]. Also, LGBT persons usually display poorer mental health compared to non-LGBT persons and it has been suggested that SU might be a coping strategy to deal with this higher burden [17, 42, 52, 56]. A study showing that adolescents living in a situation of vulnerability are more prone to SU provided further evidence for this hypothesis [51]. Multiple minority stressors and associated coping via SU in adolescence seem to contribute to the development of SUD among some LGBTQ young adults [18]. Studies showed that the higher prevalence of tobacco and alcohol in SMY were associated with, and partially explained by social stressors as bullying/victimization [7, 15, 31, 41]. On the other hand, youths living in Switzerland today have recently experienced major social changes, with marriage for all being enacted by the electorate in September 2021 with 64.1% of the vote. A Dutch study showed positive effects of same-sex marriage legalization on mental health [5]. However, a Swiss report shows that sexual minorities are still more likely to experience psychological distress and mental illness than heterosexual people [20], due to various stressors, such as experiencing discriminatory behavior [30]. Beyond the stresses that most individuals experience, minority individuals may also experience specific stressors that are unique, chronic, and socially constructed, e.g., homophobic slurs in the schoolyard. Thus, as outlined in the minority stress model, the multitude of stressors that SMY face may lead to increased susceptibility to SU [35, 40].

In addition, SU in SMY must also be understood as part of a subcultural context: Two studies in Australia showed that sexual minority youths agreed to the statement that the LGBT community has more liberal attitudes towards SU [12] and that the participation in the LGBT community is associated with higher SU involvement among sexual minority youth [13]. This has been shown in particular among men having sex with men (MSM) for sexualized SU [25, 46]. Knowing these factors (subcultural context, norms, behavior, perception) is important for efficient and successful prevention [12]. Consequently, risk and protective factors for SU among sexual minority youths should be identified, and it should be studied whether they differ from their heterosexual counterparts [11].

There are well-known demographic factors that influence SU, such as sex and gender. The representation of one's own gender, especially in adolescence, also occurs through SU as a form of gender-specific expression. One example of this is young men, who may demonstrate their masculinity in their peer group through the consumption of alcohol [33, 50]. In this context, exposure to friends’ SU is of importance: a study showed that sexual minority youths reported more willingness than non-sexual minority youths to use substances offered by peers [19]. Another study also showed the relevance of impulsivity for binge drinking to avoid subjective negative experiences, but also in the face of positive emotional experiences in young MSM within their peer group [43].

Nevertheless, many studies mentioned above have the disadvantage of selective recruitment such as medical centers [15], LGBT festival attendees [26], or via LGBT organizations [30]. None of them have utilized data from a general population cohort that has been followed-up longitudinally. Furthermore, previous research has often not examined whether substance use differs between sexual identities and across gender [11] or focuses particularly on SU by MSM because such use is perceived to be associated with sexual risk behaviors and poorer mental health [4, 23, 25]. Taken together, there is little current knowledge about the prevalence of SU (tobacco, alcohol, cannabis, Ecstasy/MDMA, stimulants, prescription opioids, benzodiazepines, hallucinogens) in sexual minority youths (SMY) compared to heterosexual youth (HET) and about how sex as well as sociodemographic and psychosocial factors are modulating this prevalence.

Thus, this study aims to answer the question of whether SU differs between SMY and HET. Firstly, we compare the 12-months prevalence rates and frequency of SU in male and female SMY compared to their HET counterparts at ages 17 and 20 in an urban Swiss cohort. Secondly, we evaluate demographic and psychosocial predictors of SU. These variables (e.g., education level, internalizing symptoms) were selected based on their previously shown relevance for substance use disparities or based on previously shown differences between SMY and HET.

## Methods

### Participants

The data used in this study was retrieved from the longitudinal and community-representative *z-proso* study [44]. Participants were selected using a cluster-stratified randomized sampling approach. In 2004, a cohort of 1,675 children was built from 56 primary classes randomly selected from 90 public schools in the city of Zurich, Switzerland’s largest city. Stratification was achieved under consideration of the school sizes and socioeconomic backgrounds of the school districts. The sample was largely representative of first graders attending public school in the city of Zurich. The participants were assessed nine times between 2005 (age 7) and 2022 (age 24). The current study uses data collected at ages 17 (n = 1297), and 20 (n = 1177).

This study is consistent with national and international ethical standards and was approved by the Ethics Committee of the Faculty of Arts and Social Sciences of the University of Zurich. Participants provided written informed consent for their study participation. Data were collected in groups of 5–25 participants in classroom settings with paper-and-pencil questionnaires at age 17 and in a laboratory setting with computer-administered surveys at age 20. Completing the surveys typically took about 90 min. Adolescents and young adults received a participation compensation of 60 CHF at age 17 and 75 CHF at age 20.

### Variables

#### Demographic variables

##### Sex

Sex is defined as biological sex assigned at birth.

##### Sexual minority youth

Sexual minority status is defined as individuals who are attracted to people of the same gender (CDC, 2021). We defined SMY as people that are also, but not exclusively, attracted to the same sex. HET is defined as youth that is attracted exclusively to the opposite sex. The question was asked for the first time in 2015, at age 17. Evaluations about sexual attraction are, therefore, only possible with data from assessments at age 17 and age 20. Participants were asked the following question: "People differ in the sexual attraction that they feel towards others. How would you describe your sexual orientation? Please indicate the statement that best describes you. With “men”, we also mean “boys”, with “women”, we also mean “girls”. Please only mark one answer." Individuals provided their ratings on a 5-point Likert scale (1 = ”I am attracted only to men”, 2 = ”I'm attracted mainly to men, but sometimes to women, too”, 3 = ”I am attracted to men and women equally”, 4 = “I am attracted mainly by women, but sometimes also by men”, 5 = ”I am attracted only to women”). Sexual minority status was identified through the participants’ sex assigned at birth and their sexual attraction. For further analyses, the answers were dichotomized (0 = exclusively heterosexual attraction [classified as HET] vs. 1 = non-exclusive same sex attraction [classified as SMY]). Individuals who did not disclose their sexuality were excluded from the study (at age 17: n = 9, at age 20: n = 3).

##### Socio-economic status

The socioeconomic status was measured by the International Socio-Economic Index of Occupational Status (ISEI), which combines income and education to reflect the status of an occupation. The participants provided information about their parents. The socioeconomic status in this analysis therefore refers to the status of the parents, not of the adolescents [8].

##### Parental migration background

Parental migration background is a binary variable indicating whether both parents were born outside of Switzerland or not. Participants reported their parents’ place of birth.

##### Education level

The participant’s education levels were drawn from the survey conducted at age 15, before completing their compulsory education. In the present study, they were divided into two groups showing a similar equidistance: High school, secondary school A or equivalent (group 1) vs. secondary school B/C, special needs class or equivalent (group 2).

#### Substance use (outcome variables)

As this study aimed to detect differences between SU of different social groups rather than SU itself, substances were grouped into categories according to their general psychopharmacological mechanism of action (see Table 1 and [57]). In these categories, each person was assigned to the consumption quantity where the highest value of consumption for any of the queried substances within one group was reported.

**Table 1:** Substance categories

Aggregation	Asked at age 17	Asked at age 20
Tobacco	Cigarettes, tobacco, shisha	Cigarettes, tobacco, shisha
Alcohol	beer, wine, mixed drinks	beer, wine, mixed drinks
	Schnapps, vodka, whisky	Schnapps, vodka, whisky
Cannabis	Hashish, weed, cannabis, marijuana	Hashish, weed, cannabis, marijuana
	n/a	synthetic cannabinoids (e.g., cannabis substitutes such as "Dutch Orange", "Spice", "K2", "Ganja Style")
Ecstasy/MDMA	Ecstasy, MDMA	Ecstasy, MDMA
Stimulants	Amphetamine, Speed, Pepp, Ice, Crystal Meth, Methamphetamine	Amphetamine or methamphetamine (e.g., "Speed", "Pepp", "Ice", "Crystal Meth")
	Cocaine	Cocaine
Opioids	n/a	Cough syrups, pastilles or drops with codeine (e.g., Resyl plus®, Makatussin®, Pectocalmine N®, Codeine Knoll®)
	n/a	Opiate painkillers (e.g., Tramal®, Co-Dafalgan®, Sevredol®/Sevre-Long®, Subutex®, Oxycontin®, Palladon®, Durogesic®)
Benzodiazepines	n/a	Tranquillisers with benzodiazepines (e.g., Temesta®, Valium®, Rohypnol®, Xanax®, Dormicum®)
Hallucinogens	LSD, Psilocybe, magic mushrooms	LSD, Psilocybe, magic mushrooms
	n/a	2C-B or other "2C drugs" (e.g., "Bromo", "Erox", "Nexus", "Venus")
	n/a	Ketamine

In the survey, the participants were asked how often they had used a substance during the previous 12 months (excluding use of medications prescribed by a physician). Assessments were made on a six-point scale: 1 = ”never”, 2 = ”once”, 3 = ”2–5 times”, 4 = ”6–12 times (monthly)”, 5 = ”13–52 times (weekly)”, and 6 = ”53–365 times (daily)”. For measuring prevalence, we grouped the answers to used substances into three categories (aligning with [47]: “never” (1), “occasional” (2, 3, and 4) and “frequent” (5 and 6). Substances that fell under the category “frequent” in less than 10 participants were dichotomized to ensure the anonymity of the participants to “no-use” (1) and “use” (2, 3, 4, 5, and 6). For the following regression analyses, all substances were dichotomized to “no-use” vs. “use”.

#### Psychosocial variables

All variables were self-reported by participants who completed a variety of questionnaires at age 17 or 20, except sensation-seeking, which was assessed at age 7. For the variables, the composite score was created from the mean value of the individual items; see Table 2 for reliability statistics.

**Table 2:** Baseline characteristics at age 17 and 20

Variable		Total % (n) or M (SD)	Males % (n) or M (SD)				Females % (n) or M (SD)			
Demographics	Age		HET		SMY		HET		SMY	
**Sexual Minority Youth** n = 1297 Missing: 9 (0.69%)	17	11.3% (147)	92.8%	605	7.2%	47	84.5%	545	15.5%	100
	20	19.4% (228)	88.1%	512	11.9%	69	73.3%	437	26.7%	159
**Socio-economic status (ISEI)** Range: 16–90, n = 1232, Missing: 65 (5%)	17	46.43 (19.43)	46.48	19.95	56.09	16.09	44.56	18.57	51.45	20.21
	20	47.13 (19.69)	46.66	19.94	57.18	18.99	43.92	18.97	52.90	18.59
**Parental migration background** both parents born abroad), n = 1267, Missing: 30 (2.3%)	17	48.3% (612)	48.2%	284	28.3%	13	53.4%	285	30.6%	30
	20	47.7% (550)	49.2%	246	34.3%	23	55.0%	238	28.1%	43
**Higher education level** n = 1268, Missing: 29 (2.2%)	17	65.6% (832)	60.3%	359	73.9%	34	66.9%	355	87.5%	84
	20	68.0% (781)	62.2%	313	77.9%	53	66.4%	283	86.8%	132

Covariates										
**Sensation-seeking** Range: 0–1 (composite score), n = 1130, Missing: 167 (12.9%)	17	0.57 (0.25)	0.68	0.22	0.60	0.21	0.47	0.22	0.46	0.27
	20	0.57 (0.25)	0.67	0.22	0.64	0.24	0.46	0.22	0.47	0.25
**Low self-control** Range: 1(fully untrue)-4(fully true), 10 Items, Cronbach’s Alpha = 0.73, n = 1239, Missing: 58 (4.5%)	17	2.21 (0.43)	2.29	0.42	2.06	0.48	2.14	0.42	2.26	0.38
	20	2.07 (0.42)	2.14	0.44	2.03	0.42	2.01	0.40	2.00	0.39
**Internalizing symptoms** Range: 1(never)-5(very often), 9 Items, Cronbach’s Alpha = 0.85, n = 1281, Missing: 16 (1.2%)	17	2.28 (0.75)	1.95	0.58	2.39	0.71	2.51	0.73	2.94	0.81
	20	2.21 (0.76)	1.96	0.64	2.31	0.71	2.34	0.78	2.64	0.79
**Bullying victimization** Range: 1(never)-6((almost) daily), 4 Items, Cronbach’s Alpha = 0.69, n = 1289, Missing: 8 (0.6%)	17	1.45 (0.57)	1.45	0.59	1.75	0.67	1.40	0.52	1.54	0.63
	20	1.37 (0.47)	1.36	0.45	1.63	0.61	1.31	0.44	1.46	0.52
**Leisure activities** Range: 1(never)-6((almost) daily), 31 Items, Cronbach’s Alpha = 0.78, n = 1152, Missing: 145 (11.2%)	17	2.23 (0.43)	2.29	0.44	2.10	0.47	2.17	0.42	2.26	0.41
	20	2.21 (0.43)	2.26	0.46	2.24	0.44	2.14	0.41	2.19	0.41
**Exposure to friends’ substance use** without alcohol and tobacco, 1 = yes, n = 1206, Missing: 91 (7.0%)	17	59.9% (772)	62.9%	342	64.3%	27	52.5%	275	81.3%	78
	20	63.4% (712)	65.5%	313	83.1%	54	51.6%	220	81.2%	125
**Self-reported subjective stress** Range: 1(never)-5(very often), 4 Items, Cronbach’s Alpha = 0.85, n = 1177, Missing: 0	17	n/a	n/a		n/a		n/a		n/a	
	20	2.82 (0.93)	2.56	0.85	3.04	0.89	2.93	0.94	3.25	0.94

##### Sensation-seeking

The behavioral measure was asked on an adapted 9-item version of the Travel Game [1, 36]. Sensation-seeking is a composite score with a value from 0 to 1, whereby a higher score indicates a higher level of sensation-seeking.

##### Low self-control

The 10 items covered five dimensions: risk-seeking, impulsivity, self-centeredness, preference for physical activity, and short-temperedness [21]. Responses were recorded on a four-point Likert scale (1 = fully untrue, 2 = somewhat untrue, 3 = somewhat true, 4 = fully true). Higher scores indicate lower levels of self-control.

##### Internalizing symptoms

The 9 items of the Social Behavior Questionnaire [38] focus on questions on crying, fear, or feeling alone. Answers were provided on a five-point Likert scale (1 = never, 2 = rarely, 3 = sometimes, 4 = often, 5 = very often). Higher scores indicated higher levels of internalizing symptoms.

##### Bullying victimization

The 4 items focused on questions about being ignored, insulted, physically attacked and personal belongings being hidden or stolen. A fifth item on sexual bullying was dropped from the composite scale [37]. Responses were recorded on a six-point scale (1 = never, 2 = few times a year, 3 = about once per month, 4 = about once per week, 5 = 2–3 times per week, 6 = (almost) daily). Higher scores indicated higher levels of bullying victimization.

##### Leisure activities

The 31 items focused on the frequency of going out, meeting friends, participating in group events, or practicing sports. Responses were recorded on a six-point scale (1 = never, 2 = few times a year, 3 = about once per month, 4 = about once per week, 5 = 2–3 times per week, 6 = (almost) daily). Higher scores indicated more frequent leisure activities. The items consist of separate subdimensions. To obtain a relative score of leisure activities that can be compared between the studied groups, they were combined into a single composite score for this study.

##### Exposure to friends’ illegal substance use

Participants were asked to indicate their two best friends and their intimate partner and to report on their illegal SU (e.g., cannabis, cocaine) on a binary scale. The composite binary score indicates whether at least one of the three friends had used illegal substances in the past 12 months.

##### Self-reported subjective stress

This variable was only assessed at age 20. The 4 items focused on the feelings of control, stress, and overcoming difficulties. Answers were provided on a five-point Likert scale (1 = never, 2 = rarely, 3 = sometimes, 4 = often, 5 = very often). Higher scores indicated higher levels of subjective stress.

### Analytic strategy

In a first step, SMY and HET were compared in terms of their baseline characteristics and SU, stratified by sex (males, females). Wherever appropriate, Cronbach's alpha was used to calculate whether an item affects inter-item correlation.

Differences in demographic variables between the groups were tested for significance at both ages. We checked the associations of psychosocial variables by running logistic or linear regressions, depending on whether the variable is categorical (= logistic) or continuous (= linear).

In a second step, a block-wise regression at both ages was performed: Firstly, a univariate logistic regression examined associations between SMY-status and SU. We compared results for the following groups of participants and at both ages: HET and SMY, HET-males and SMY-males and HET-females and SMY-females. Secondly, a multivariate logistic analysis served to include demographic variables as covariates. Parental migration background was already a binary variable, ISEI was split into two groups at the median to identify a group-related over- or under-average. Finally, the analysis was run again with the integration of all psychosocial variables as covariates to identify whether effects are still significant after adding demographic or psychosocial variables into the model.

## Results

### Demographics

Of the survey respondents aged 17, 50.3% were male and 49.7% female. Of the respondents aged 20, 48.9% were male and 51.1% female. There was no self-reported intersex person in the sample. Several demographic variables differed significantly between SMY and HET (see Table 2).

#### Sexual minority status

While 0.69% of the respondents did not disclose their sexual attraction at age 17, only 0.25% refrained from it at age 20. At age 17, 7.2% of males and 15.5% of females reported same-sex sexual attraction, percentages which increased to 11.9% and 26.7% respectively in the sample aged 20.

#### Socio-economic status

Reporting SMY status was associated with higher socioeconomic status. At age 17, the mean ISEI was higher for SMY-males (56.1) than HET-males (44.6). This was also the case for SMY-females (51.5) compared to HET-females (44.6). At age 20, the differences remained in males (SMY: 57.2, HET: 46.7) and females (SMY: 52.9, HET: 43.9). U-tests revealed that the ISEI differed significantly (p < 0.001) in all comparisons.

#### Parental migration background

SMY were more likely to have two parents born in Switzerland than HET: 48.2% of HET-males in the sample aged 17 had a parental migration background, compared to merely 28.3% of SMY-males (Chi-square test: p = 0.009). Similar numbers resulted for females (53.4% as opposed to 30.6% respectively, p < 0.001). At age 20, the differences persisted: 49.2% HET-males reported parental migration background as opposed to 34.3% SMY-males (p = 0.022 and so did 55.0% of HET-females compared to 28.1% of SMY-females, p < 0.001).

#### Education level

Reporting SMY status was partially associated with higher levels of education. At age 17, 60.3% of HET-males compared to 73.9% of SMY-males had a higher level of education, which was not significantly different (Chi-square test: p = 0.069). Conversely, for female respondents groups differed significantly (p < 0.001) with values of 66.9% and 87.5% for HET versus SMY-females, respectively.

At age 20, a similar picture emerged: 62.2% of HET-males enjoyed a higher level of education as opposed to 77.9% of SMY-males (p = 0.01). A slightly wider gap was visible for females with 66.4% of HET-females compared to 86.6% of SMY-females benefitting from higher education (p < 0.001).

### Covariates

Linear regressions showed that sexual minority was associated with higher levels of internalizing symptoms, both at ages 17 and 20 (see Additional file 1: Table S1). SMY-males reported significantly higher levels of internalizing symptoms at ages 17 (β = 0.45, p < 0.001) and 20 (β = 0.34, p < 0.001), and so did SMY-females at ages 17 (β = 0.44, p < 0.001) and 20 (β = 0.30, p < 0.001).

Similarly, sexual minority status was associated with higher levels of bullying victimization, both at ages 17 and 20. SMY-males reported significantly higher levels of bullying victimization at ages 17 (β = 0.30, p = 0.001) and 20 (β = 0.27, p < 0.001), and so did SMY-females at ages 17 (β = 0.14 p = 0.020) and 20 (β = 0.15, p = 0.001).

SMY-males reported fewer leisure activities (β = − 0.19, p = 0.005) at age 17 and less sensation-seeking (β = − 0.08, p = 0.03) than HET-males, whereby these differences were no longer significant at age 20. The levels of self-control among SMY-males increased from β = − 0.02 (p = 0.001) to − 0.11 (p = 0.044) between ages 17 and 20. Furthermore, logistic regressions showed that at age 17, SMY-males were less likely to be exposed to a friend’s substance use (excl. tobacco and alcohol) than HET-males (OR = 0.73, p = 0.31). Conversely, their likelihood of exposure at age 20 was 2.6 times higher than for HET-males (p = 0.006). This indicates a reversal of the effect within 3 years.

SMY-females were not significantly different from HET-females in terms of their leisure activities or sensation-seeking. However, at age 17, SMY-females reported lower levels of self-control (β = 0.12, p = 0.01), a value that realigned between SMY and HET-females at age 20. Moreover, SMY-females have an approximately 4-times higher likelihood of being exposed to a friend’s SU excl. tobacco and alcohol at both, ages 17 and 20 (p < 0.001).

At age 20, both SMY-males (linear regression coefficient β = 0.48) and SMY-females (β = 0.32) reported significantly (p < 0.001) higher levels of subjective stress than their HET counterparts.

### Substance use

**Fig. 1:**
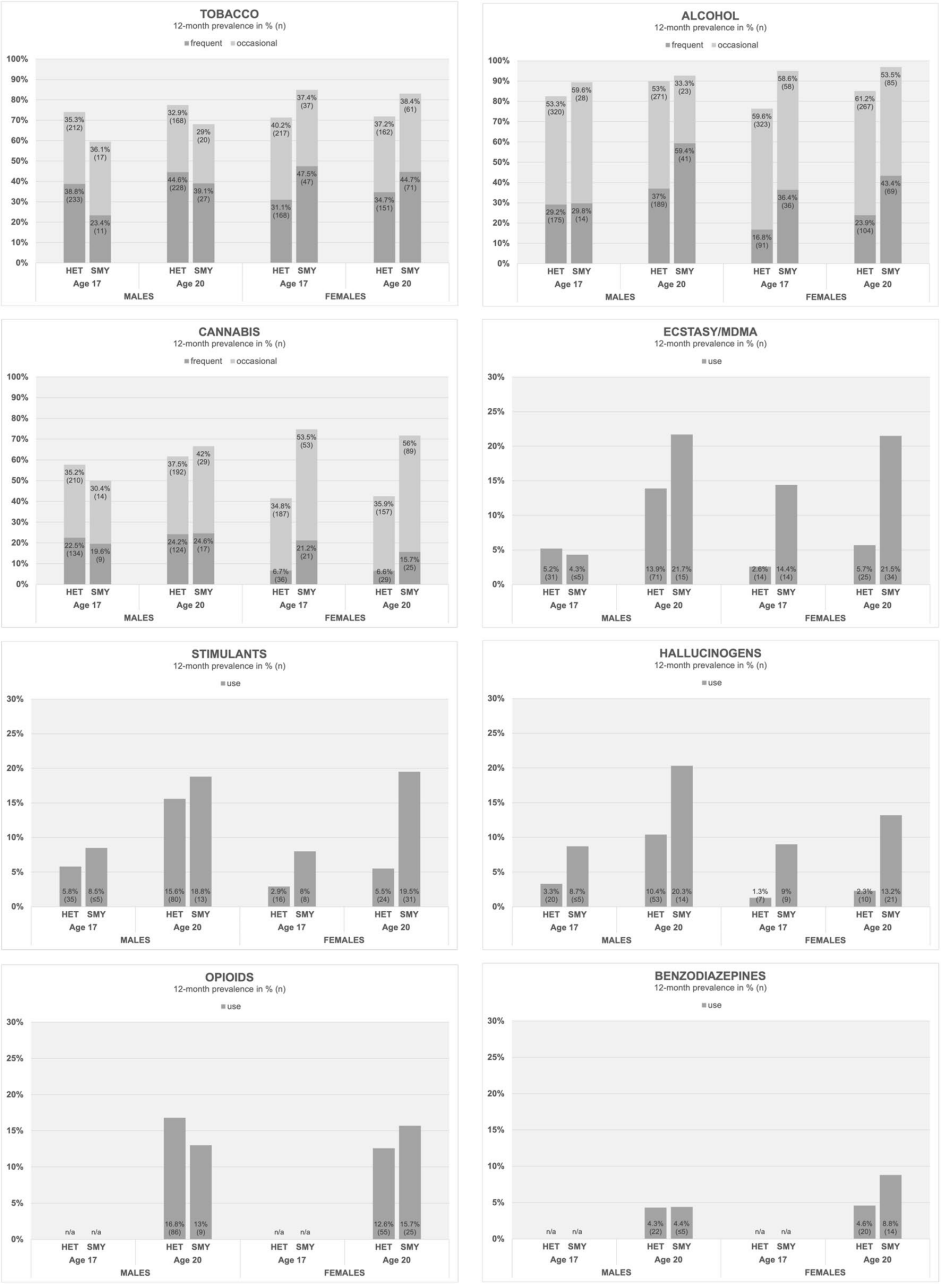
Substance use at age 17 and 20 (12-month prevalence)

Overall, SMY- and HET-youth differed in their 12-month prevalence of SU. The systematic stratification by sex revealed differences within SMY-males and SMY-females compared to the HET-groups (see Figure 1 and Additional file 1: Table S2). SMY-females reported higher rates of use (vs. no use) and frequent use (vs. occasional/never) for all examined substances compared to HET-females both at age 17 and 20. This was not the case for SMY-males compared to HET-males: SMY-males reported lower rates of frequent (vs. occasional/never) tobacco use at age 17 and 20; higher rates of frequent alcohol and cannabis use at age 17 and 20; a shift from lower to higher rates of use (vs. no use) of Ecstasy/MDMA from age 17 to 20; and higher rates of use of stimulants and hallucinogens both at age 17 and 20.

#### Males

The univariate logistic regression showed associations between SMY und SU (see Additional file 1: Table S3). Among males, only two significant differences could be found: at age 17, SMY-males used less tobacco than HET-males (OR = 0.51, p = 0.03), while at age 20, SMY-males used more hallucinogens than HET-males (OR = 2.20, p = 0.02).

When adjusted for demographic variables in a multivariate logistic regression (see Additional file 1: Table S4), 17-year-old SMY-males showed significantly lower levels of tobacco (aOR = 0.49, p = 0.03) and cannabis use (aOR = 0.45, p = 0.02) at age 17. At age 20, SMY-males used significantly more Ecstasy/MDMA (aOR = 2.00, p = 0.04) and hallucinogens (aOR = 2.30, p = 0.02).

When adjusted for demographics and psychosocial variables (see Table 3 for ages 17 and 20), there were significantly higher use-levels for SMY-males compared to HET-males of both Ecstasy/MDMA (aOR = 2.30, p = 0.04) and hallucinogens (aOR = 2.43, p = 0.03) at age 20.

**Table 3:** Associations among SMY and SU at age 17 and 20, adjusted for demographic and psychosocial variables

Substance no-use vs. use		SMY reference: HET			SMY-males reference: HET-males			SMY-females reference: HET-females		
	Age	OR	95% CI	p value	OR	95% CI	p value	OR	95% CI	p value
Tobacco	17	0.99	0.56–1.75	0.98	0.72	0.30–1.72	0.46	1.21	0.54–2.72	0.65
	20	1.28	0.82–1.99	0.28	0.57	0.29–1.13	0.11	**2.06**	**1.09–3.89**	**0.03**
Alcohol	17	1.91	0.68–5.38	0.22	3.42	0.38–30.92	0.27	1.45	0.44–4.81	0.54
	20	1.63	0.65–4.1	0.30	1.45	0.3–6.99	0.65	2.06	0.61–6.92	0.24
Cannabis	17	1.34	0.77–2.33	0.29	0.58	0.22–1.49	0.26	**2.14**	**1.04–4.38**	**0.04**
	20	1.48	0.96–2.27	0.07	0.84	0.41–1.70	0.62	**2.24**	**1.28–3.91**	**0.004**
Ecstasy/MDMA	17	**2.93** ^a^	**1.22–7.07**	**0.02**	0.81 ^a^	0.09–7.81	0.86	**4.29** ^a^	**1.40–13.15**	**0.01**
	20	**2.70**	**1.65–4.44**	**< 0.001**	**2.30**	**1.06–5.00**	**0.04**	**3.93**	**1.88–8.2**	**< 0.001**
Stimulants	17	**2.46** ^a^	**0.99–6.12**	**0.05**	3.07 ^a^	0.66–14.16	0.15	2.64 ^a^	0.75–9.29	0.13
	20	**1.74**	**1.05–2.88**	**0.03**	1.18	0.54–2.59	0.67	**3.45**	**1.58–7.53**	**0.002**
Hallucinogens	17	**5.11** ^a^	**1.98–13.22**	**< 0.001**	4.22 ^a^	0.87–20.47	0.07	**5.59** ^a^	**1.39–22.49**	**0.02**
	20	**2.86**	**1.63–5.00**	**< 0.001**	**2.43**	**1.10–5.37**	**0.03**	**6.65** ^a^	**2.34–18.88**	**< 0.001**
Opioids	17	n/a			n/a			n/a		
	20	0.78	0.46–1.3	0.33	0.79	0.34–1.83	0.59	0.83	0.42–1.65	0.59
Benzodiazepines	17	n/a			n/a			n/a		
	20	1.14	0.54–2.39	0.73	0.96	0.25–3.71	0.95	1.91	0.71–5.16	0.20

Highlighted in bold is p ≤ 0.05. A logistic regression was run with adjustments for socio-economic status, parental migration background, education level, sensation-seeking, low self-control, internalizing symptoms, bullying, leisure activities, exposure to friends’ substance use, and self-reported subjective stress.

^a^Due to collinearity of own substance use and exposure to a friends' substance use, only those observations that have exposure to friends' substance use were examined

#### Females

The univariate logistic regression showed that SMY-females had higher rates of SU than HET-females for almost every substance examined (see Additional file 1: Table S3). The highest OR was shown for hallucinogens at age 17 (OR = 7.56, p < 0.001) and the lowest significant OR for tobacco at age 20 (OR = 1.91, p = 0.006).

When adjusted for demographic variables in a multivariate logistic regression, SMY-females showed significantly higher levels of SU for all substances examined except opioids at age 20.

When adjusted for demographics and psychosocial variables (see Table 3 for ages 17 and 20), there were significant higher levels for the use of cannabis (aOR = 2.14, p = 0.04), Ecstasy/MDMA (aOR = 4.29, p = 0.01) and hallucinogens (aOR = 5.59, p = 0.02) at age 17. At age 20, SMY-females reported significantly higher rates of SU than HET-females for tobacco (aOR = 2.06, p = 0.03), cannabis (aOR = 2.24, p = 0.004), Ecstasy/MDMA (aOR = 3.93, p < 0.001), stimulants (aOR = 3.45, p = 0.002), and hallucinogens (aOR = 6.65, p < 0.001).

## Discussion

This study showed clear differences between SMY and HET in their 12-month prevalence of SU. The difference cannot be explained by the studied demographics and psychosocial variables alone. Conversely, prevalence figures may be misleading because SMY-status is associated with numerous psychosocial and demographic variables.

When adjusting for those variables, SMY-males showed significantly higher levels of use of Ecstasy/MDMA and hallucinogens at age 20 compared to HET-males. SMY-females compared to HET-females showed significantly higher levels of use of cannabis, Ecstasy/MDMA, and hallucinogens at age 17 and 20 and higher levels of use of tobacco and stimulants at age 20. A significantly lower SU in SMY-individuals could not be found for any substance after the adjustment for several confounders. Thus, this study contributes insights into at what age, and how, SU begins to differentiate by SMY-status. The effect direction and size differ in the within-sex comparison and depend on the substance examined. Therefore, they must be discussed for every group and every substance separately.

Reporting belonging to SMY is significantly associated with a higher socioeconomic status, a Swiss (as opposed to a migration) history of parents and higher levels of education. SMY also have significant higher rates of internalizing symptoms (at age 20: β = 0.40), bullying victimization (β = 0.17), self-reported subjective stress (β = 0.49), and exposure to friends’ substance use (OR = 3.11) than HET. SMY-males have a higher level of self-control and lower levels of sensation-seeking and leisure activities than HET-males at age 17, which was less pronounced at age 20. In contrast, SMY-females revealed lower level of self-control than HET-females at the age of 17.

With multivariate regression analysis integrating demographics and psychosocial variables, we could show that higher levels of SU are associated with several characteristics previously described, such as internalizing symptoms or bullying victimization. These results are particularly robust because the data was collected within the same group in a long-term cohort with a high-quality data assessment. Of note, the difference can only be partially explained by psychosocial variables expected to be related to sexual minority status.

However, not all differences can be explained with the integration of these factors into the regression model, as shown by the aOR. The examined groups still showed several significant differences in SU. The prevalence of SU of SMY-females is on the level of HET-males or even exceeds this. After adjusting for demographic and psychosocial factors, the odds remained significantly higher for almost all substances. Hence, difference in SU is established earlier and is more pronounced in SMY-females at a young age.

After adjustment for demographics and psychosocial variables, SMY-males show at age 17 significant lower rates for tobacco and cannabis than HET-males. However, at age 20, they show significant higher levels for the use of Ecstasy/MDMA and hallucinogens.

Many group comparisons in males were not significant. It remains open whether differences are indeed not existing, or whether the statistical power was too low because of the smaller N of the male SMY compared to the female SMY group. Also, a ceiling effect is conceivable, given that the difference between HET-male and SMY-male becomes relatively smaller, even if the level of SU of these groups are higher compared to females. This has been shown internationally with differences in SU that were larger among female groups than male groups [11]. Nevertheless, it is surprising that SMY-males use less than HET-males at the age of 17 but catch up this difference within three years. Differences can be seen in SMY-females compared to HET-females: even when numerous variables are considered, significant differences remained stable.

The strength and direction of the change of association between SMY-status and SU by the inclusion of demographic and psychosocial variables also provided indications of their significance for the respective substance: For tobacco, for example, the integration of confounders leads to a reduction of the difference between SMY and the HET comparison group. Remarkably, for ecstasy/MDMA the opposite phenomenon occurs, where the integration of confounders increases the difference between SMY and HET (i.e. a greater effect is seen with higher OR of SMY-male SU at age 20 when including psychosocial and demographic factors in a multivariate regression).

Already at a young age, experiences of belonging to a sexual minority seem to be associated with more SU possibly as a behavioral response to psychological and social-environmental factors. Especially in the comparison between SMY-males and SMY-females, it becomes clear how the examined psychosocial factors can explain effects: SMY-males have a higher level of self-control than HET-males at age 17. In contrast, at the age of 17, SMY-females have a lower level of self-control than HET-females. Therefore, self-control, which is also associated to belonging to SMY, even though gender-specific, may play an important role in explaining differences of SU.

SMY-females also have significantly higher exposure to friends’ SU at age 17 compared to HET-females than SMY-males compared to HET-males. With SMY-males, exposure to friends’ SU increases within three years compared to HET males (at age 17: OR = 0.73, p = 0.31; at age 20: 2.59, p = 0.006).

Taken together, these results strengthen the importance of the investigated psychosocial variables for explaining differences in SU between adolescents when we consider the pronounced differences between SMY-males and SMY-females. Moreover, the results also suggest that not all findings can be explained by the analyzed covariates.

### Limitations

Our prospective-longitudinal cohort study has many strengths, but also comes with some limitations. First, the associations among SU and SMY are based on correlational data, thus, direction of effects or causation cannot be inferred. Second, it must be assumed that not all people have reported their SMY status. Although there are less than 1% that did not disclose sexual attraction, it is possible that answers were given according to social desirability. Both underreporting of exclusive same-sex attraction and overreporting of sexual openness are conceivable and shown in self-reported sexual behaviors and even under anonymous responding with evidence that extreme under- or over-reporting is as common as is found in other fields [27]. To distinguish from this are individuals that do not consciously make a false statement, but don’t feel sexual attraction to the same gender for any possible individual, cultural, or social reasons. This could explain differences between men and women in the proportion of SMY [29], but this is not necessarily due to any bias. Third, self-reports underestimated the prevalence of most substances by 30–60% compared to hair tests [49]. How young people report SU and whether the internalized view of parents/teachers or peers rather cause under- or over-reporting, must be carefully considered when interpreting results. Fourth, the data only provides information on sexual attraction, but not on sexual identity or sexual practices. Same-sex attraction might overestimate the prevalence of LGB in youth, as same-sex attraction does not necessarily go along with self-identification as a member of the LGB community and same-sex behavior [20]. Fifth, we assigned participants as male or female using their sex assigned at birth. However, participants were not asked as part of the questionnaire whether they identify with this at the time of data collection. Data did not allow any conclusions to be drawn about gender minority youth (e.g., trans or non-binary youth). Sixth, there are substances that are particularly prevalent in the gay subculture [22, 39, 55] that were not assessed in this survey, notably gamma-hydroxybutyric acid/gamma-butyrolactone (GHB/GBL) and alkyl nitrites (“poppers”).

## Conclusions

This study highlights the importance of considering sexuality- and sex-based differences in SU. Both SMY-males and SMY-females are different from their gender majority, especially associations among SMY-females and SU even after adjusted for demographic and psychosocial variables are significantly different.

Differences between HET and SMY are not only evident in the data regarding SU, but also regarding demographic and psychosocial factors. For example, our analyses show that ISEI and parental migration background, but also various stressors such as bullying victimization, are significantly associated with SMY status. A study conducted with the same data showed that a lower ISEI or a parental migration background were associated with an decreased risk of SU [48]. However, neither the differences in demographic nor psychosocial factors may explain all the variance found in our models showing differences in SU. Thus, both demographic and psychosocial effects may combine with specific sociocultural influences. Some studies have pointed out that the sexualized use of substances among the gay men culture plays an important role [14, 22, 25]. Other studies emphasize the importance of gender expression through SU in young people [33, 50]. Further research is needed here to gain a more nuanced understanding of the different demographic, psychosocial and subcultural factors that contribute to SU among SMY.

SU is an important and urgent area in public health in which LGBT persons are disadvantaged compared to the rest of the population in Switzerland [30]. It has been shown that minority stressors and associated coping via SU contribute to SUD among some SMY [18]. Our findings underline the importance of incorporating LGBT-sensitive as well as LGBT-specific perspectives in research, policy, prevention, intervention, and treatment [2, 10, 11, 28, 30, 53]. It must be considered that SU not only has health consequences, but may have perceived positive effects for the individual, especially in a subcultural context. Therefore, it is important not to contribute to perpetuations of stigma and social exclusion based on substance use and sexual practices [45]. This is equally true for belonging to a sexual minority. Belonging to a sexual minority can also be a source of special resources (e.g., resilience [23] and preventive factors [54]) concerning SU and secondly, SU can also be an expression of a certain attitude towards life and not a coping strategy, e.g., openness to new experiences, especially sexual.

In this context, it is important to examine not only health deficits but also health-promoting factors among minorities (e.g., individual resilience, social networks). In this study, for example, it appears that between the age of 17 and 20, internalizing symptoms among SMY decrease, but reporting of bullying victimization remains the same. This could be interpreted as an expression of resilience. Further research is needed here. Any public health measures must promote these LGBTQ-specific, preventive factors, to be effective. It is especially important to take SMY seriously in their sexual identity at an early age, which can be associated with certain patterns of SU [6]. In dialogue with the communities, the social function of SU in younger years for SMY-females and in later adolescence for SMY-males must be addressed.

### Supplementary Information

**Additional file 1: Table S1.** Associations among SMY-status, sex, and psychosocial variables at age 17 and 20. **Table S2.** Substance use at age 17 and 20 (12-month prevalence). **Table S3.** Associations among SMY and SU at age 17 and age 20. **Table S4.** Associations among SMY and SU at age 17 and 20, adjusted for demographic variables.

## Data Availability

The data that support the findings of this study are available from Jacobs Center for Productive Youth Development at University of Zurich, but restrictions apply to the availability of these data, which were used under license for the current study, and so are not publicly available. Data are however available from the authors upon reasonable request and with permission of University of Zurich.

## Abbreviations

aOR: Adjusted odds ratio
HET: Heterosexual youth
ISEI: International socio-economic index of occupational status
LGBTQ: Lesbian, gay, bisexual, transgender, queer
MDMA: 3,4-Methylendioxy-N-methylamphetamin
MSM: Men having sex with men
OR: Odds ratio
SMY: Sexual minority youth
SU: Substance use
SUD: Substance use disorder
