# Supplementary material for: Substance use in sexual minority youth: prevalence in an urban cohort
Source: Child Adolesc Psychiatry Ment Health. 2023 Sep 16;17:109. doi: 10.1186/s13034-023-00657-0 (PMC10505308; doi:10.1186/s13034-023-00657-0)
Supplement: Supplementary file 1 — Additional file 1: Table S1. Associations among SMY-status, sex, and psychosocial variables at age 17 and 20. Table S2. Substance use at age 17 and 20 (12-month prevalence). Table S3. Associations among SMY and SU at age 17 and age 20. Table S4. Associations among SMY and SU at age 17 and 20, adjusted for demographic variables. [file 13034_2023_657_MOESM1_ESM.docx]

***Substance use in sexual minority youth: prevalence in an urban cohort***

**Additional material**

**Additional Table 1: Associations among SMY-status, sex, and psychosocial variables at age 17 and 20**

| **Variable** | | **SMY**  Reference: HET | | | **SMY-males**  Reference: HET-males | | | **SMY-females**  Reference: HET-females | | |
| --- | --- | --- | --- | --- | --- | --- | --- | --- | --- | --- |
|  | **Age** | β^a^  OR^b^ | 95%CI | *p value* | β^a^  OR^b^ | 95%CI | *p value* | β^a^  OR^b^ | 95%CI | *p value* |
| **Sensation  Seeking** ^a^ | **17** | **-0.07** | **-0.12- -0.02** | ***.002*** | **-0.08** | **-0.15- -0.01** | ***.031*** | -0.01 | -0.06-0.44 | *.74* |
|  | **20** | **-0.06** | **-0.09- -0.02** | ***.004*** | -0.03 | -0.09-0.03 | *.341* | 0.01 | -0.04-0.05 | *.920* |
| **Low Self-control** ^a^ | **17** | -0.02 | -0.10-0.05 | *.60* | **-0.02** | **-0.36- -0.09** | ***.001*** | **0.12** | **0.03-0.21** | ***.01*** |
|  | **20** | **-0.07** | **-0.14- -0.01** | ***.018*** | **-0.11** | **-0.22- -0.01** | ***.044*** | -0.01 | -0.09-0.06 | *.704* |
| **Internalizing symptoms** ^a^ | **17** | **0.56** | **0.43-0.68** | ***<.001*** | **0.45** | **0.27-0.63** | ***<.001*** | **0.44** | **0.28-0.60** | ***<.001*** |
|  | **20** | **0.40** | **0.29-0.50** | ***<.001*** | **0.34** | **0.18-0.51** | ***<.001*** | **0.30** | **0.15-0.43** | ***<.001*** |
| **Bullying**  **Victimization** ^a^ | **17** | **0.18** | **0.08-0.28** | ***<.001*** | **0.30** | **0.12-0.48** | ***.001*** | **0.14** | **0.02-0.25** | ***.020*** |
|  | **20** | **0.17** | **0.10-0.24** | ***<.00*** | **0.27** | **0.15-0.38** | ***<.001*** | **0.15** | **0.06-0.23** | ***.001*** |
| **Leisure activities** ^a^ | **17** | -0.03 | -0.10-0.05 | *.45* | **-0.19** | **-0.23- -0.58** | ***.005*** | 0.09 | -0.01-0.18 | *.066* |
|  | **20** | -0.01 | -0.06-0.06 | *.973* | -0.02 | -0.14-0.09 | *.697* | 0.05 | -0.02-0.12 | *.184* |
| **Exposure Friends’ Substance Use** ^b^ | **17** | **2.02** | **1.40-2.91** | ***<.001*** | 0.73 | 0.40-1.34 | *.31* | **4.18** | **2.57-6.79** | ***<.001*** |
|  | **20** | **3.11** | **2.16-4.50** | ***<.001*** | **2.59** | **1.32-5.08** | ***.006*** | **4.04** | **2.58-6.31** | ***<.001*** |
| **Self-reported subjective stress** ^a^ | **17** | *n/a* | | | *n/a* | | | *n/a* | | |
|  | **20** | **0.49** | **0.33-0.59** | ***<.001*** | **0.48** | **0.27-0.69** | ***<.001*** | **0.32** | **0.14-0.49** | ***<.001*** |

Highlighted in bold is p≤.05; ^a^ standardized linear regression coefficient β for linear regressions; ^b^ odds ratios for logistic regressions.

**Additional Table 2: Substance use at age 17 and 20 (12-month prevalence)**

| **Variable**  *occasional = once – monthly, frequent = weekly – daily, no use = never, use = once – daily* | | | **TOTAL** % (n) | **MALES** % (n) | | | | **FEMALES** % (n) | | | |
| --- | --- | --- | --- | --- | --- | --- | --- | --- | --- | --- | --- |
|  |  |  |  | **HET %** | **n** | **SMY**  **%** | **n** | **HET %** | **n** | **SMY**  **%** | **n** |
| **Tobacco** | **Age 17**  n=1286  Missing: 0.9% | **never** | 26.8% (344) | 25.8% | 155 | 40.4% | 19 | 28.7% | 155 | 15.2% | 15 |
|  |  | **occasional** | 37.6% (483) | 35.3% | 212 | 36.1% | 17 | 40.2% | 217 | 37.4% | 37 |
|  |  | **frequent** | 35.7% 459) | 38.8% | 233 | 23.4% | 11 | 31.1% | 168 | 47.5% | 47 |
|  | **Age 20**  n=1174  Missing: 0.3% | **never** | 24.4% (286) | 22.5% | 115 | 31.9% | 22 | 28.1% | 122 | 17% | 27 |
|  |  | **occasional** | 35.0% (411) | 32.9% | 168 | 29% | 20 | 37.2% | 162 | 38.4% | 61 |
|  |  | **frequent** | 40.6% (477) | 44.6% | 228 | 39.1% | 27 | 34.7% | 151 | 44.7% | 71 |
| **Alcohol** | **Age 17**  n=1288  Missing: 0.7% | **never** | 18.9% (243) | 17.5% | 105 | 10.6% | ≤5 | 23.6% | 128 | 5.1% | ≤5 |
|  |  | **occasional** | 56.6% (729) | 53.3% | 320 | 59.6% | 28 | 59.6% | 323 | 58.6% | 58 |
|  |  | **frequent** | 24.5% (316) | 29.2% | 175 | 29.8% | 14 | 16.8% | 91 | 36.4% | 36 |
|  | **Age 20**  n=1175  Missing: 0.2% | **never** | 10.7% (126) | 10% | 51 | 7.3% | 5 | 14.9% | 65 | 3.1% | 5 |
|  |  | **occasional** | 55.0% (646) | 53% | 271 | 33.3% | 23 | 61.2% | 267 | 53.5% | 85 |
|  |  | **frequent** | 34.3% (403) | 37% | 189 | 59.4% | 41 | 23.9% | 104 | 43.4% | 69 |
| **Cannabis** | **Age 17**  n=1279  Missing: 1.4% | **never** | 48.1% (615) | 42.3% | 252 | 50.0% | 23 | 58.6% | 315 | 25.3% | 25 |
|  |  | **occasional** | 36.3% (464) | 35.2% | 210 | 30.4% | 14 | 34.8% | 187 | 53.5% | 53 |
|  |  | **frequent** | 15.6% (200) | 22.5% | 134 | 19.6% | 9 | 6.7% | 36 | 21.2% | 21 |
|  | **Age 20**  n=1177  Missing: 0.0% | **never** | 43.8% (515) | 38.3% | 196 | 33.3% | 23 | 57.4% | 251 | 28.3% | 45 |
|  |  | **occasional** | 39.7% (467) | 37.5% | 192 | 42% | 29 | 35.9% | 157 | 56% | 89 |
|  |  | **frequent** | 16.6% (195) | 24.2% | 124 | 24.6% | 17 | 6.6% | 29 | 15.7% | 25 |
| **Ecstasy/MDMA** | **Age 17**  n=1286  Missing: 0.9% | **no use** | 95.3% (1225) | 94.8% | 569 | 95.7% | 45 | 97.4% | 528 | 85.6% | 83 |
|  |  | **use** | 4.7% (61) | 5.2% | 31 | 4.3% | ≤5 | 2.6% | 14 | 14.4% | 14 |
|  | **Age 20**  n=1173  Missing: 0.3% | **no use** | 87.6% (1028) | 86.1% | 439 | 78.3% | 54 | 94.3% | 411 | 78.5% | 124 |
|  |  | **use** | 12.4% (145) | 13.9% | 71 | 21.7% | 15 | 5.7% | 25 | 21.5% | 34 |
| **Stimulants** | **Age 17**  n=1292  Missing: 0.4% | **no use** | 95.1% (1229) | 94.2% | 566 | 91.5% | 43 | 97.1% | 528 | 92% | 92 |
|  |  | **use** | 4.9% (63) | 5.8% | 35 | 8.5% | ≤5 | 2.9% | 16 | 8.0% | 8 |
|  | **Age 20**  n=1177  Missing: 0.0% | **no use** | 87.4% (1029) | 84.4% | 432 | 81.2% | 56 | 94.5% | 413 | 80.5% | 128 |
|  |  | **use** | 12.6% (148) | 15.6% | 80 | 18.8% | 13 | 5.5% | 24 | 19.5% | 31 |
| **Hallucinogens** | **Age 17**  n=1287  Missing: 0.8% | **no use** | 96.9% (1247) | 96.7% | 579 | 91.3% | 42 | 98.7% | 535 | 91.0% | 91 |
|  |  | **use** | 3.1% (40) | 3.3% | 20 | 8.7% | ≤5 | 1.3% | 7 | 9.0% | 9 |
|  | **Age 20**  n=1177  Missing: 0.0% | **no use** | 91.7% (1079) | 89.7% | 459 | 79.7% | 55 | 97.7% | 427 | 86.8% | 138 |
|  |  | **use** | 8.3% (98) | 10.4% | 53 | 20.3% | 14 | 2.3% | 10 | 13.2% | 21 |
| **Opiods** | **Age 17** | **n/a** | | | | | | | | | |
|  | **Age 20**  n=1176  Missing: 0.1% | **no use** | 85.1% (1001) | 83.2% | 425 | 87% | 60 | 87.4% | 382 | 84.3% | 134 |
|  |  | **use** | 14.9% (175) | 16.8% | 86 | 13% | 9 | 12.6% | 55 | 15.7% | 25 |
| **Benzodizepines** | **Age 17** | **n/a** | | | | | | | | | |
|  | **Age 20**  n=1176  Missing: 0.1% | **no use** | 95.0% (1117) | 95.7% | 489 | 95.7% | 66 | 95.4% | 417 | 91.2% | 145 |
|  |  | **use** | 5.0% (59) | 4.3% | 22 | 4.4% | ≤5 | 4.6% | 20 | 8.8% | 14 |

**Additional Table 3: Associations among SMY and SU at age 17 and age 20**

| **Substance**  no-use vs. use |  | **SMY**  Reference: HET | | | **SMY-males**  Reference: HET-males | | | **SMY-females**  Reference: HET-females | | |
| --- | --- | --- | --- | --- | --- | --- | --- | --- | --- | --- |
|  | **Age** | OR | 95%CI | *p value* | OR | 95%CI | *p value* | OR | 95%CI | *p value* |
| **Tobacco** | **17** | 1.23 | 0.82-1.85 | *.32* | **0.51** | **0.29-0.95** | ***.03*** | **2.25** | **1.26-4.03** | ***.006*** |
|  | **20** | 1.22 | 0.86-1.73 | *.26* | 0.62 | 0.36-1.07 | *.09* | **1.91** | **1.20-3.03** | ***.006*** |
| **Alcohol** | **17** | **3.49** | **1.81-6.73** | ***<.001*** | 1.78 | 0.69-4.61 | *.23* | **5.81** | **2.31-14.60** | ***<.001*** |
|  | **20** | **3.04** | **1.57-5.90** | ***.001*** | 1.42 | 0.55-3.69 | *.47* | **5.40** | **2.13-13.66** | ***<.001*** |
| **Cannabis** | **17** | **2.02** | **1.40-2.91** | ***<.001*** | 0.73 | 0.40-1.34 | *.31* | **4.18** | **2.57-6.79** | ***<.001*** |
|  | **20** | **2.16** | **1.58-2.97** | ***<.001*** | 1.25 | 0.74-2.16 | *.40* | **3.57** | **2.40-5.32** | ***<.001*** |
| **Ecstasy/MDMA** | **17** | **3.05** | **1.67-5.55** | ***<.001*** | 0.82 | 0.19-3.52 | *.79* | **6.36** | **2.93-13.82** | ***<.001*** |
|  | **20** | **2.44** | **1.67-3.56** | ***<.001*** | 1.72 | 0.92-3.21 | *.09* | **4.51** | **2.59-7.84** | ***<.001*** |
| **Stimulants** | **17** | 1.91 | 0.99-3.67 | *.053* | 1.50 | 0.51-4.43 | *.46* | **2.87** | **1.19-6.90** | ***.018*** |
|  | **20** | **1.94** | **1.32-2.86** | ***.001*** | 1.25 | 0.66-2.40 | *.45* | **4.17** | **2.36-7.36** | ***<.001*** |
| **Hallucinogens** | **17** | **4.03** | **2.03-8.01** | ***<.001*** | 2.76 | 0.91-8.44 | *.08* | **7.56** | **2.75-20.80** | ***<.001*** |
|  | **20** | **2.55** | **1.64-3.97** | ***<.001*** | **2.20** | **1.15-4.23** | ***.02*** | **6.50** | **2.99-14.13** | ***<.001*** |
| **Opioids** | **17** | *n/a* | | | *n/a* | | | *n/a* | | |
|  | **20** | 1.00 | 0.67-1.51 | *.99* | 0.74 | 0.35-1.55 | *.43* | 1.30 | 0.78-2.16 | *.32* |
| **Benzodiazepines** | **17** | *n/a* | | | *n/a* | | | *n/a* | | |
|  | **20** | 1.74 | 0.97-3.11 | *.063* | 1.01 | 0.29-3.47 | *.99* | 2.01 | 0.99-4.09 | *.053* |

Highlighted in bold is p≤.05.

**Additional Table 4: Associations among SMY and SU at age 17 and 20, adjusted for demographic variables**

| **Substance**  no-use vs. use |  | **SMY**  Reference: HET | | | **SMY-males**  Reference: HET-males | | | **SMY-females**  Reference: HET-females | | |
| --- | --- | --- | --- | --- | --- | --- | --- | --- | --- | --- |
|  | **Age** | OR | 95%CI | *p value* | OR | 95%CI | *p value* | OR | 95%CI | *p value* |
| **Tobacco** | **17** | 1.16 | 0.76-1.76 | *.50* | **0.49** | **0.26-0.94** | ***.03*** | **2.11** | **1.16-3.85** | ***.01*** |
|  | **20** | 1.33 | 0.92-1.94 | *.13* | 0.66 | 0.37-1.17 | *.15* | **2.07** | **1.25-3.45** | ***.01*** |
| **Alcohol** | **17** | 1.78 | 0.89-3.56 | *.10* | 0.85 | 0.30-2.40 | *.77* | **3.06** | **1.17-8.00** | ***.02*** |
|  | **20** | 1.61 | 0.8-3.21 | *.18* | 0.79 | 0.29-2.15 | *.64* | **2.79** | **1.06-7.34** | ***.03*** |
| **Cannabis** | **17** | 1.33 | 0.90-1.96 | *.15* | **0.45** | **0.24-0.87** | ***.02*** | **2.89** | **1.72-4.83** | ***<.001*** |
|  | **20** | **1.67** | **1.19-2.33** | ***.003*** | 1.03 | 0.58-1.83 | *.91* | **2.57** | **1.68-3.93** | ***<.001*** |
| **Ecstasy/MDMA** | **17** | **2.36** | **1.20-4.61** | ***.01*** | 0.88 | 0.20-3.91 | *.87* | **4.37** | **1.80-10.61** | ***<.001*** |
|  | **20** | **2.45** | **1.63-3.69** | ***<.001*** | **2.00** | **1.03-3.86** | ***.04*** | **3.62** | **1.99-6.57** | ***<.001*** |
| **Stimulants** | **17** | 1.91 | 0.94-3.87 | *.07* | 1.75 | 0.57-5.36 | *.33* | **2.71** | **1.00-7.31** | ***.05*** |
|  | **20** | **1.90** | **1.25-2.9** | ***.003*** | 1.39 | 0.71-2.75 | *.34* | **3.57** | **1.9-6.7** | ***<.001*** |
| **Hallucinogens** | **17** | **3.40** | **1.63-7.09** | ***<.01*** | 2.45 | 0.78-7.75 | *.13* | **6.14** | **1.96-19.24** | ***<.001*** |
|  | **20** | **2.37** | **1.48-3.78** | ***<.001*** | **2.30** | **1.17-4.54** | ***.02*** | **5.17** | **2.22-12.04** | ***<.001*** |
| **Opioids** | **17** | *n/a* | | | *n/a* | | | *n/a* | | |
|  | **20** | 1.00 | 0.65-1.55 | *.995* | 0.83 | 0.39-1.76 | *.62* | 1.18 | 0.67-2.07 | *.57* |
| **Benzodiazepines** | **17** | *n/a* | | | *n/a* | | | *n/a* | | |
|  | **20** | **2.01** | **1.07-3.79** | ***.03*** | 1.11 | 0.31-3.95 | *.88* | **2.66** | **1.2-5.91** | ***.02*** |

Highlighted in bold is p≤.05. A logistic regression was run with adjustments for socio-economic status, parental migration background and education level.
